# Biochemical, pharmacological, and toxicological attributes of caper (*Capparis ovata*) flowering buds and berries pickles

**DOI:** 10.1002/fsn3.3012

**Published:** 2022-07-30

**Authors:** Ozden Ozgun‐Acar, Gurbet Celik‐Turgut, Hüseyin Guner, Serdar Sezer, Alaattin Sen

**Affiliations:** ^1^ Seed Breeding & Genetics Application Research Center Pamukkale University Denizli Turkey; ^2^ Organic Agriculture Management, Faculty of Applied Sciences Pamukkale University Denizli Turkey; ^3^ Department of Molecular Biology and Genetics, Faculty of Life and Natural Sciences Abdullah Gul University Kayseri Turkey; ^4^ Institute of Chemical Technology Marmara Research Center, TUBITAK Kocaeli Turkey; ^5^ Department of Pharmacology, Faculty of Medicine Suleyman Demirel University Isparta Turkey; ^6^ Department of Biology, Faculty of Arts & Sciences Pamukkale University Denizli Turkey

**Keywords:** anti‐inflammatory, *Capparis ovata*, complementary, toxicology, whole‐genome transcriptome

## Abstract

*Capparis ovata* is a natural plant that grows widely in Turkey and its flowering buds and berry pickle are used in traditional medicine. Thus, the current study was expanded to evaluate the biochemical, pharmacological, and toxicological aspects of the *Capparis ovata* water extract (COWE). To determine the biochemical properties of COWE, mineral and fatty acid content, elemental analysis, flavonoid/phenolic content, radical‐scavenging capacity, and pesticide analysis were performed. Furthermore, to find out whether it had anti‐inflammatory properties, reverse transcription‐polymerase chain reaction (RT‐PCR) and nuclear factor kappa B (NF‐κB) luciferase activity tests were conducted. Whole‐genome transcriptomic profiling was carried out at a dose level of 500 mg/kg COWE to understand its pharmacological effect. Transaminases in serum were tested, and quantitative polymerase chain reaction (qPCR) was done using a custom design array that included the stress and molecular toxicology pathway to establish its toxicological qualities. As a result of the evaluations, it was observed that COWE has a high mineral and unsaturated fatty acid content, flavonoid/phenolic content, and radical‐scavenging ability. It significantly inhibited NF‐κB transcriptional activity as well as inflammatory cytokine expression in T‐lymphoblast cells. Whole‐genome transcriptomic profiling depicted that COWE modulates immune responses by upregulating natural killer cell activation, cellular response to type I interferon, B‐cell proliferation and differentiation, and Janus kinase–signal transducer and activator of transcription (JAK–STAT) pathways. Molecular Toxicology Pathfinder RT^2^ Profiler PCR array analysis revealed that COWE at or lower dose of 500 mg/kg/day did not cause a comparatively adverse effect. According to the findings, COWE is a rich source of nutrients and can be used as an adjunct therapy for various inflammatory diseases.

## Graphical abstract

The current study with detailed analyses strongly suggested that caper (*Capparis ovata*) is a rich source of nutrients and can be used as an adjunct therapy for various inflammatory and autoimmune diseases.

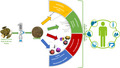

## 1 INTRODUCTION

Plant products have become increasingly popular in recent years, particularly in the Western world, where they are frequently used in conjunction with conventional therapeutic medications. Studies suggest that diet, nutrition, herbal and natural products interventions could play a significant role in disease prevention, management, treatment, and even as therapeutics in some circumstances (Downer et al., 2020; Kim et al., 2018; Medellín‐Luna et al., 2019). On the other hand, during the last decade, unequivocal evidence has been presented that the constituents in food supplements may be substrates, inhibitors, or inducers of various xenobiotic/drug‐metabolizing enzymes and have an impact on the pharmacokinetics of any co‐administered drugs metabolized by this system (Koziolek et al., 2019; Wanwimolruk & Prachayasittikul, 2014).

The caper is a native Mediterranean plant, and certain species of caper have been cultivated as an economically significant plant (Duman & Özcan, 2014). It has been reported that the genus *Capparis* consists of nearly 80 species. *Capparis ovata* and *Capparis spinosa* have wide natural distribution in Turkey, and they are consumed as pickles (Davis, 1965). In general, Capparaceae family members contain glucosinolates, alkaloids, and flavonoids (Matthäus & Özcan, 2002, 2005; Siracusa et al., 2011). The fatty acid composition of the *C. ovata* seeds contains oleic, linoleic, and palmitic acids (Matthäus & Özcan, 2005). Caper species have traditionally been used for rheumatism, diabetics, and kidney infections and caper buds, root bark, and berries have shown a number of potential pharmacological activities such as anti‐inflammatory, anti‐allergic, anti‐apoptotic, antimicrobial, antioxidant, and immunomodulatory effects (Bilen et al., 2016; Boongapim et al., 2021; Ozgun‐Acar et al., 2016; Yu et al., 2017). Recent research has revealed that *C. ovata* may have anti–neuroinflammatory properties that could be useful in treating neuroinflammatory disorders such as Alzheimer’s and multiple sclerosis (Ozgun‐Acar et al., 2016). Despite being such a widely used plant in traditional medicine, there is no comprehensive study on the biochemical, pharmacological, and toxicological properties of *C. ovata* pickles. In addition, there is no definite information about the dose that people should use in the literature. As a result, the current study was expanded to investigate and assess the biochemical, pharmacological, and toxicological features of water extract of *C. ovata* pickles, as this is the most common and traditionally used form throughout the Mediterranean, and to determine the appropriate doses to be used.

## 2 MATERIALS AND METHODS

### 2.1 Preparation of lyophilized COWE

The pickles of *C. ovata* flowering buds and berries were supplied by Asci Murat Capers, Ice Cream, Dessert and Pickle Manufacturing & Export Co., Ltd., used for the preparation of aqueous extracts, and then lyophilized as described previously (Ozgun‐Acar et al., 2016). Briefly, 7.75 g of flower buds and 2.25 g of fruit of *C. ovata* pickles were air‐dried in the shade and crushed into a fine powder at a mortar, extracted with 100 ml of distilled water using a fully automatic rapid extraction system (C. Gerhardt Analytical Systems), and then lyophilized at −105°C (Labconco Freeze Dryer).

### 2.2 Biochemical analysis

The total protein content of the extract was determined by the Kjeldahl Method as described by the Association of Official Agricultural Chemists International (AOAC 2000 955.04). The color of the extract was analyzed as defined by the Commission Internationale de l'Eclairage (CIE). The sample is dissolved to homogeneity in the high‐speed blender and is placed in the reading room of the calorimeter device to determine CIE L*, a*, b*, C* (Chroma), and ho (hue angle) values. The CIE L* a* b*, color system represents quantitative relationship of colors on three axes: L* value indicates lightness, and a* and b* are chromaticity coordinates. Ten grams of the extract was placed in a beaker and dissolved in about three times the ultrapure, distilled–deionized water (30 ml) to give a suitable consistency, and heated in a water bath (37°C) for 30 min while stirring with a glass rod. The product is then milled with a blender, and the pH value is determined by the pH meter device. The moisture content of the sample was determined in a vacuum drying cabinet (Lee & Kim, 2009).

As it is the first step for elemental analysis, the percentage ash content of the extract was determined by a gradual burning at 550°C until the residual whites were obtained after performing a preburn process within alcohol. The microwave digestion of the samples was performed with 65% nitric acid (HNO_3_) and 1.5 ml of 30% hydrogen peroxide (H_2_O_2_) microwave reaction system using seven steps of 7 min with the following power: 150, 250, 300, 400, 500, and 600 W (vent, Tmax = 180°C). The samples were analyzed using a Thermo Scientific iCAP™ RQ ICP‐MS system with a micromist nebulizer (Al‐Hakkani, 2019).

The concentration of total flavonoid content in the COWE was calculated from the standard plot and expressed as mg quercetin equivalent (QE)/g of dried plant material (Quettier‐Deleu et al., 2000). The determination of total phenolic content was estimated spectrophotometrically according to the Folin–Ciocalteu colorimetric method and expressed as milligram equivalents of gallic acid (GAE)/g (Singleton et al., 1999). Total oil content determination was performed according to ISO 659:1998, and fresh oil was analyzed for fatty acids. Fatty acid methyl esters were determined according to the method specified in ISO 12966‐2:2017. Residual pesticide analysis of the COWE was determined applying the multiresidue methodology separating analytes from one another, so that they are measured using either gas chromatography–mass spectrometry (GC–MS) or high‐performance liquid chromatography–mass spectrometry (HPLC–MS) according to guidelines defined by the Food and Drug Administration (FDA; Hayward et al., 2013).

### 2.3 Pharmacological analysis

The C57BL/6 female mice (18–22 g, 12 weeks) were purchased from Kobay Experimental Animals Laboratory (Ankara, Turkey) and housed at the University Animal House in standard conditions and fed a standard pellet diet with water ad libitum. All experimental procedures in animals were performed under appropriate regimes with veterinary services within licensed projects approved by the Institutional Experimental Animal Ethics Committee (PAUHDEK‐2012/005). The mice were randomly assorted into four groups as Control, COWE_250_, COWE_500_, and COWE_750_. Each group consisted of eight mice, and they received 250, 500, and 750 mg COWE per kg body weight intragastrically for 28 days while control subjects received only water. At the end of the experimental period, the animals were killed after 16 h of fasting. Blood samples were taken from the aorta to determine the serum enzymes, and the livers were removed, rinsed with cold physiological saline, and stored at −80°C until analyzed.

To evaluate hepatocellular damage and cytotoxic potential of the COWE, enzymatic leakage of transaminases [alanine aminotransferase (ALT) and aspartate aminotransferase (AST)], lactate dehydrogenase (LDH), and gamma‐glutamyl transferase (GGT) in serum was measured by the methods of Reitman and Frankel (1957), Wroblewski and Ladue (1955) and Orlowski and Meister (1963), respectively (Orlowski & Meister, 1963; Reitman & Frankel, 1957; Wroblewski & Ladue, 1955). The serum biochemistry data for the normal range were gathered at the Mouse Phenome Database at The Jackson Laboratory (https://phenome.jax.org/).

Antioxidant activity of the plant extracts was determined based on the radical‐scavenging effect of 1,1‐dipheny1‐2‐picrylhydrazyl (DPPH)‐free radical‐scavenging activity (Semiz et al., 2016).

To assess the anti‐inflammatory effect of the COWE on the transcriptional activity of nuclear factor kappa B (NF‐κB), CCRF‐CEM (ATCC, CCL‐119) cells were transfected with Fugene HD transfection reagent using pGL4.32 [luc2P/NF‐κB‐RE/Hygro] plasmid containing five copies of an NF‐κB response element (NF‐κB‐RE) that drives transcription of the luciferase reporter gene luc2P. Cells were stimulated with 2 μg/ml lipopolysaccharide (LPS). After 24‐h incubation, cells were treated with nontoxic doses of COWE and then the luciferase activity was determined by the Dual‐Glo Luciferase Assay System (Turgut et al., 2017). Nontoxic doses of COWE were determined according to the trypan blue stain and counted in an automatic cell counter (NanoEnTek; Yavuz et al., 2017). The anti‐inflammatory effects of COWE were further assessed by applying reverse transcription‐quantitative polymerase chain reaction (RT‐qPCR) to determine the messenger RNA (mRNA) expression levels of the selected inflammation‐related genes (RANTES, CXCL9, CXCL10, IL‐6, IL‐10, NF‐κB, and TNF‐α) in CCRF‐CEM (T lymphoblast) cells treated with COWE at nontoxic doses (Gazioglu et al., 2020).

Whole‐genome transcriptomic profiling was performed at 500 mg/kg COWE dose level to clarify the detailed pharmacological action of the extract using Agilent SurePrint G3 Mouse GE 8X60K Microarrays on Agilent 2100 Bioanalyzer (Agilent Technologies). Raw data were extracted using Agilent Feature Extraction Software (v11.0.1.1), transformed by logarithm, and normalized by the quantile method. All data analysis and visualization of differentially expressed genes were conducted using ExAtlas (https://lgsun.irp.nia.nih.gov/exatlas/; Sharov et al., 2015). Gene‐Enrichment and Functional Annotation analysis for significant probe list was performed using DAVID (Database for Annotation, Visualization, and Integrated Discovery; http://david.abcc.ncifcrf.gov/home.jsp; Huang et al., 2009; Sharov et al., 2015). We had the microarrays done by the DONE Genetics Ltd.

### 2.4 Toxicological analysis

Total RNA was isolated from mice livers using RNeasy Plus Universal Kit (Qiagen). The experimental procedure and the conditions for the qPCR were similar to those previously described (Ozgun‐Acar et al., 2016). A custom design array including mouse stress and molecular toxicology pathway was synthesized by Bioneer, and qPCR was performed in the Exicycler 96 Thermal Block from Bioneer. Three endogenous control genes, namely, beta‐2‐microglobulin (B2M), glyceraldehyde 3‐phosphate dehydrogenase (GAPDH), and beta‐actin (ACTB), found on the PCR array were used for data normalization. The qPCR using custom‐designed primers for the genes is listed in Table S1. Each replicate cycle threshold (*C*_t_) was normalized to the average *C*_t_ value of 3 endogenous controls per plate. Variations in the liver gene expression between control and COWE‐treated animals are shown as a fold increase or decrease.

### 2.5 Statistical analysis

Statistical analyses were performed using the Minitab 13 statistical software package (Minitab Inc.). Comparison between groups was performed using Student’s *t*‐test, and *p* ≤ .05 was selected as the level required for statistical significance. For the PCR‐array data analysis, the GeneGlobe data analysis web portal (Qiagen) was used. Normalized raw array data obtained from the chipset were log2 transformed and imported into R statistical computing platform as an expression dataset. Limma package is used for calculating differential expression patterns of conditions of interest. Gprofiler2 is implemented to convert available gene identifiers into their human orthologs. ClusterProfiler and some additional packages were utilized to analyze gene enrichment and functional analysis of differentially expressed sets of genes. Analysis workflow is developed in‐house using an R programming environment and can be provided upon request.

## 3 RESULTS AND DISCUSSION

### 3.1 Biochemical properties

Food composition and attributes are frequently determined in studies on food science and medicinal foods. An assay for moisture, ash, and protein content is among the most basic and crucial analytical processes conducted on a food extract (Pomeranz & Meloan, 1994). The lyophilized COWE is a light yellowish–greenish powder (Table 1), almost lacking a significant amount of water and quite acidic that might be considered an advantage for long‐term stability during storage. The high protein and mineral content (Tables 1 and 2) of the COWE makes it a healthy source of nutraceuticals as the plant’s crude protein, fibers, and minerals are critical in maintaining biochemical metabolism and physiological processes in humans (Das et al., 2012; Makkar et al., 2020). The COWE mineral content (Na, K, Ca, Mg, Fe, Zn) is high, whereas heavy metals (Hg, Cd, Pb, Pa, Cr) are present in trace amounts, which could be beneficial for its use as a supplement. It is known that people in arid and semiarid countries have utilized caper berry pickles since ancient times as a source of protein, carbohydrates, and vitamins because of their high nutritional value (Gupta & Sharma, 2007; Ozcan, 2005).

**TABLE 1:** Physical, chemical, biochemical, and microbial attributes of *Capparis ovata* water extract (COWE)

Parameter	Description	Figure
Color codes D65 (L*; a*; b*)	58.75; 7.29; 21.92	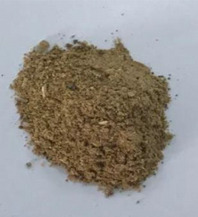
pH	4.57 ± 0.18	
Loss on drying (%)	5.9 ± 0.31	
Ash (%)	42 ± 1.15	
Protein (%)	14.19 ± 0.24	
Total flavonoids (mg QE/g)	99.7 ± 7.1	
Total phenolics (mg Ru/g)	27.33 ± 1.5	
DPPH IC50 (mg/ml)	0.25 ± 0.02	
Yeast (CFU)	<10	
Mold (CFU)	<2	
Total bacteria (CFU)	43 ± 3	

**TABLE 2:** Fatty acids and mineral composition of *Capparis ovata* water extract (COWE)

Element	mg/kg	Fatty acid	(%)
Arsenic (As)	0.5	C8:0 Caprylic Acid	0.09
Barium (Ba)	1.8	C10:0 Capric Acid	0.05
Boron (B)	18.3	C12:0 Lauric Acid	0.18
Cadmium (Cd)	<0.1	C14:0 Myristic Acid	0.31
Calcium (Ca)	2135	C14:1 Myristoleic Acid	0.01
Chromium (Cr)	0.7	C15:0 Pentadecanoic Acid	0.14
Cobalt (Co)	1.4	C15:1 cis‐Pentadecanoic Acid	0.03
Copper (Cu)	16	C16:0 Palmitic Acid	14.25
Iridium (Ir)	<0.02	C16:1 Palmitoleic Acid	0.45
Iron (Fe)	258.7	C17:0 Heptadecanoic Acid	0.39
Lead (Pb)	0.7	C18:0 Stearic Acid	6.33
Magnesium (Mg)	884	C18:1n9c Oleic Acid	17.8
Manganese (Mn)	7.7	C18:2n6 Linoleic Acid	10.1
Mercury (Hg)	<0.1	C18:3n3 a‐Linolenic Acid	23.4
Molybdenum (Mo)	4.7	C20:0 Arachidic Acid	0.95
Nickel (Ni)	22.7	C20:1 cis‐Eicosenoic Acid	8.3
Osmium (Os)	0.5	C20:2 cis‐11,14‐Eicosadienoic Acid	0.01
Palladium (Pd)	<0.1	C20:3n3 cis‐11,14,17‐Eicosatrienoic Acid	0.75
Platinum (Pt)	<0.02	C20:4n6 Arachidonic Acid	0.64
Potassium (K)	4748	C22:0 Behenic Acid	0.19
Rhodium (Rh)	<0.02	C22:2 cis‐13,16‐Docosadienoic Acid	0.1
Ruthenium (Ru)	<0.02	C24:0 Lignoceric acid	10.4
Selenium (Se)	0.3	C24:1 Nervonic Acid	0.75
Sodium (Na)	117251	C25:0 Hyenic Acid	2.9
Strontium (Sr)	51.8	C26:0 Cerotic Acid	1.2
Tin (Sn)	33.2	C28:0 Montanic Acid	0.6
Vanadium (V)	0.3		
Zinc (Zn)	21.5		

Characterizing essential fatty acid composition, a critical step for presenting edible and healthful oils, is another quality parameter for plant extracts. The results of the COWE’s fatty acid profile are shown in Table 2. The primary components were measured to be seven fatty acids, which account for over 91% of the total. Unsaturated fatty acids dominate the mixture, accounting for 62.3% of the total content, and the remaining 37.7% is saturated. Linolenic acid, an ϖ‐3 fatty acid, was discovered to be the most abundant component (23.4%), followed by oleic, linoleic, and eicosanoic acids, in that order (See Table 2). It was found that ϖ‐3 and ϖ‐6 fatty acids account for more than 51% of the unsaturated fatty acids in COWE. Thus, it is a good measure of the nutritional value of COWE vegetable oil and its human health benefits are an adequate level of linolenic (ϖ‐3) and oleic acid content (Barceló‐Coblijn & Murphy, 2009; Leikin‐Frenkel et al., 2021; Sen, 2020). Although there are many studies on the *Capparis spinosa*, a few studies have looked at the biochemical composition of *C. ovata* (Ozcan et al., 2004; Ozgun‐Acar et al., 2016). According to our results, the plant and the pickles made from flowering buds and berries are good nutrients. COWE has nutritional content that is equivalent to, if not greater than, those of many vegetables.

### 3.2 Pharmacological properties

The phenolic content of COWE was estimated by the Folin–Ciocalteu reagent and was 27.33 mg/g expressed in gallic acid equivalents (GAE). The flavonoid content of COWE was determined as 99.70 mg/g (QE; Table 1). These results indicated that the aqueous extract was found to contain a high amount of flavonoids and phenols. Plant secondary metabolites with an aromatic ring and at least one hydroxyl group are known as flavonoids and other phenolic substances, appearing as aglycones, glycosides, and methylation derivatives in plants (Tungmunnithum et al., 2018). DPPH‐free radical‐scavenging activity of COWE with an IC50 value of 0.25 mg/ml demonstrated considerable antioxidant activity that correlates well with the content of phenolic and flavonoid compounds (Table 1). These results strongly suggest that COWE might be an effective antioxidant, anticancer, anti‐inflammatory, and immune system promoting supplement, which makes it an exciting candidate for pharmaceutical and medical applications, as the vast majority of the research reported the health benefits of flavonoids and other phenolic compounds from medicinal plant species (Ahmed et al., 2016; Kumar & Pandey, 2013; Mani et al., 2021).

Prior to consumption, caper berries are typically pickled in saltwater and traditionally fermented using lactic acid bacteria (LAB; Pulido et al., 2007). Although LAB fermentation is primarily used to carry out the biological debittering process, it also significantly enhances secondary metabolites since LAB shows a high capacity to metabolize them from raw materials. For example, while quercetin is not naturally occurring in raw berries, it is found in fermented food as a by‐product of the dehydration of rutin (Jiménez‐López et al., 2018). Siracusa et al. reported that several diglycoside flavonols, such as quercetin, kaempferol, and isorhamnetin‐3‐O‐rutinoside, have previously been discovered in fermented caper berries. The health advantages of fermented caper fruits are superior due to the high variety and amount of phenolic and flavonoid substances they contain. Long‐term intake of phenolic and flavonoid substances has been proven to be protective against the development and management of diseases such as cardiovascular diseases, diabetes, osteoporosis, cancer, and neurodegenerative diseases (Mutha et al., 2021; Tungmunnithum et al., 2018). For this reason, the fermented capers and berries were used in this investigation to study their health benefits. In addition, we measured the residual pesticides in the extract to ensure that they were not polluted with environmental contaminants. We were unable to identify any of the pesticides studied (Table S1).

**FIGURE 1:**
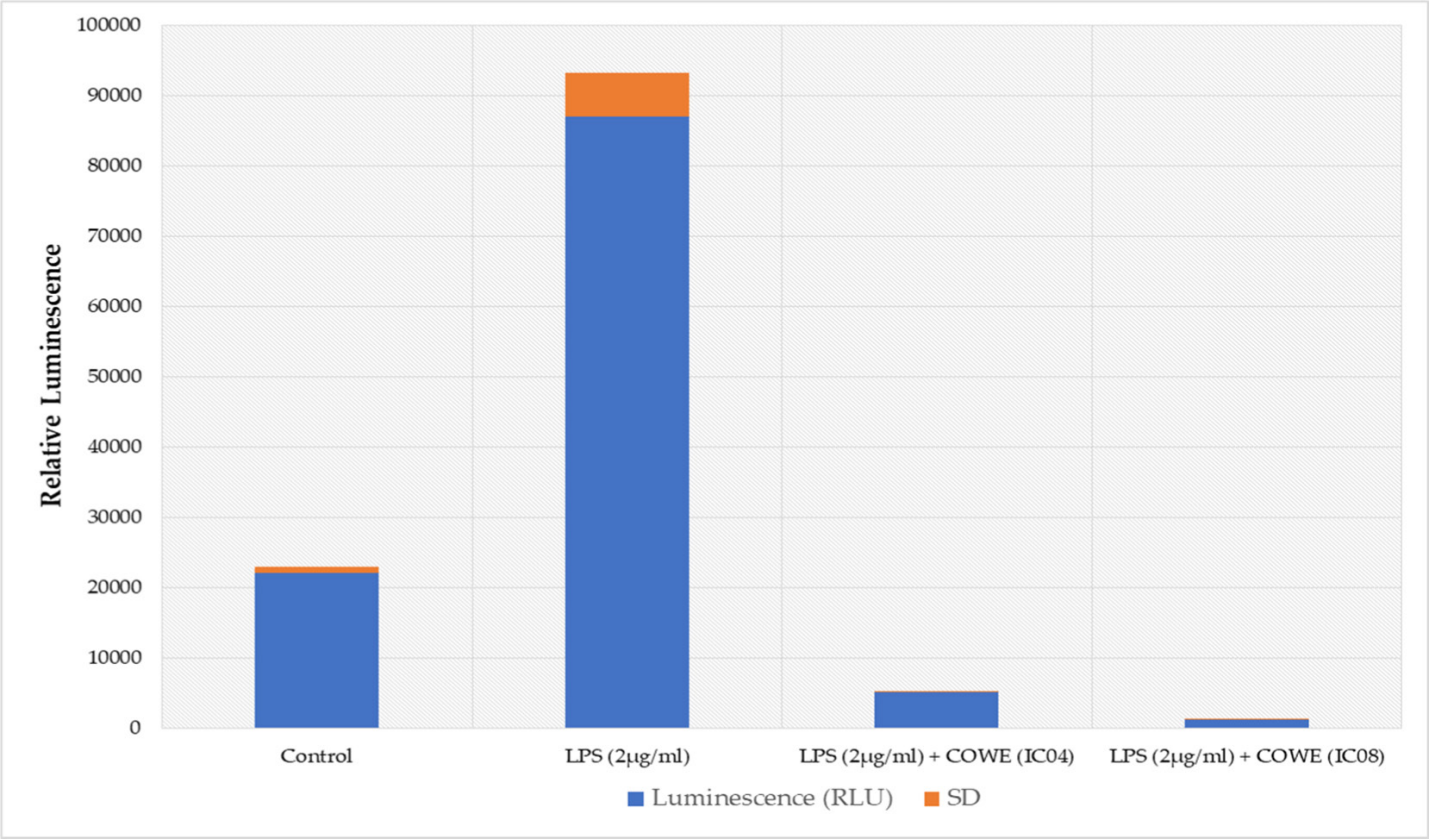
The effect of *Capparis ovata* water extract (COWE) on lipopolysaccharide (LPS)‐induced nuclear factor kappa B (NF‐κB) activation. Cells were treated as described in “Materials and methods” and NF‐κB luciferase reporter activity was then measured using dual‐glo luciferase assay system. Results are expressed as mean ± SD of three independent replicates of triplicate measurements.

In LPS‐induced CCRF‐CEM cells, NF‐κB reporter and dual‐luciferase assays were used to investigate COWE’s anti‐inflammatory impact. COWE treatment at low doses (the EC04 dose is 1.25 mg/ml and the EC08 dose is 1.5 mg/ml for CCRF‐CEM cells) dramatically reduced LPS‐induced NF‐κB transcriptional activity (Figure 1). LPS was able to promote the expression of this luciferase, which was fully inhibited by COWE treatment.

**FIGURE 2:**
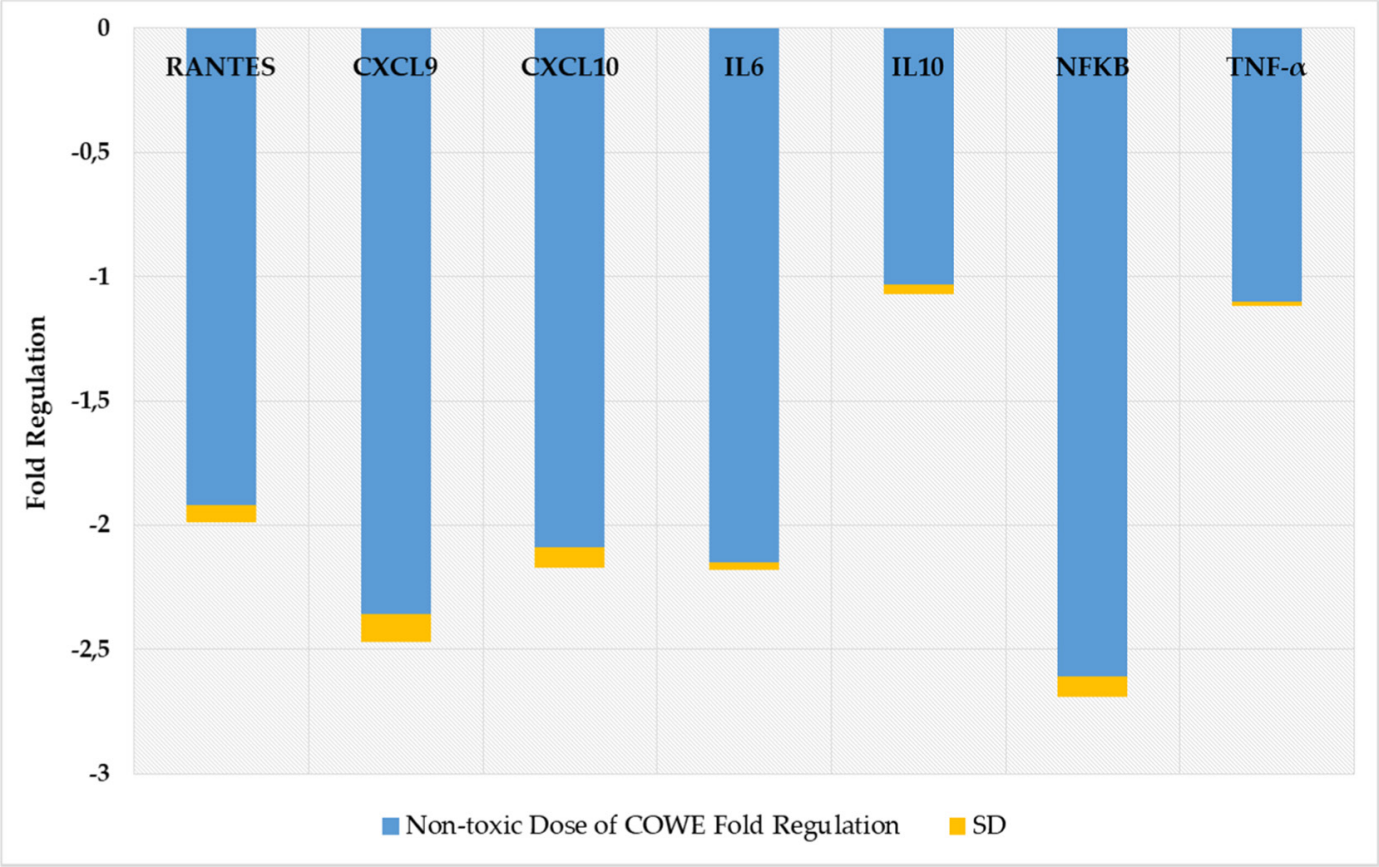
The inhibitory effect of *Capparis ovata* water extract (COWE) on inflammatory cytokines’ messenger RNA (mRNA) expression levels in CCRF‐CEM cells. Results are expressed as mean ± SD of three independent replicates of triplicate measurements.

The current study also looked at COWE treatment’s effect on the expression of inflammatory cytokine genes, such as RANTES, CXCL9, CXCL10, IL‐6, IL‐10, NF‐κB, and TNF‐α, in CCRF‐CEM cells (Figure 2). The findings revealed a significant decrease in mRNA levels of all the genes, most notably at NF‐κB, CXCL9, and IL‐6, well‐known inflammatory cytokine genes. IL‐10 is an anti‐inflammatory cytokine that controls or suppresses inflammation (Saraiva & O'Garra, 2010). Our findings indicate that COWE has a strong anti‐inflammatory effect. Thus, COWE appears to be an effective NF‐κB antagonist that inhibits NF‐κB related inflammatory pathways in T‐cells.

**FIGURE 3:**
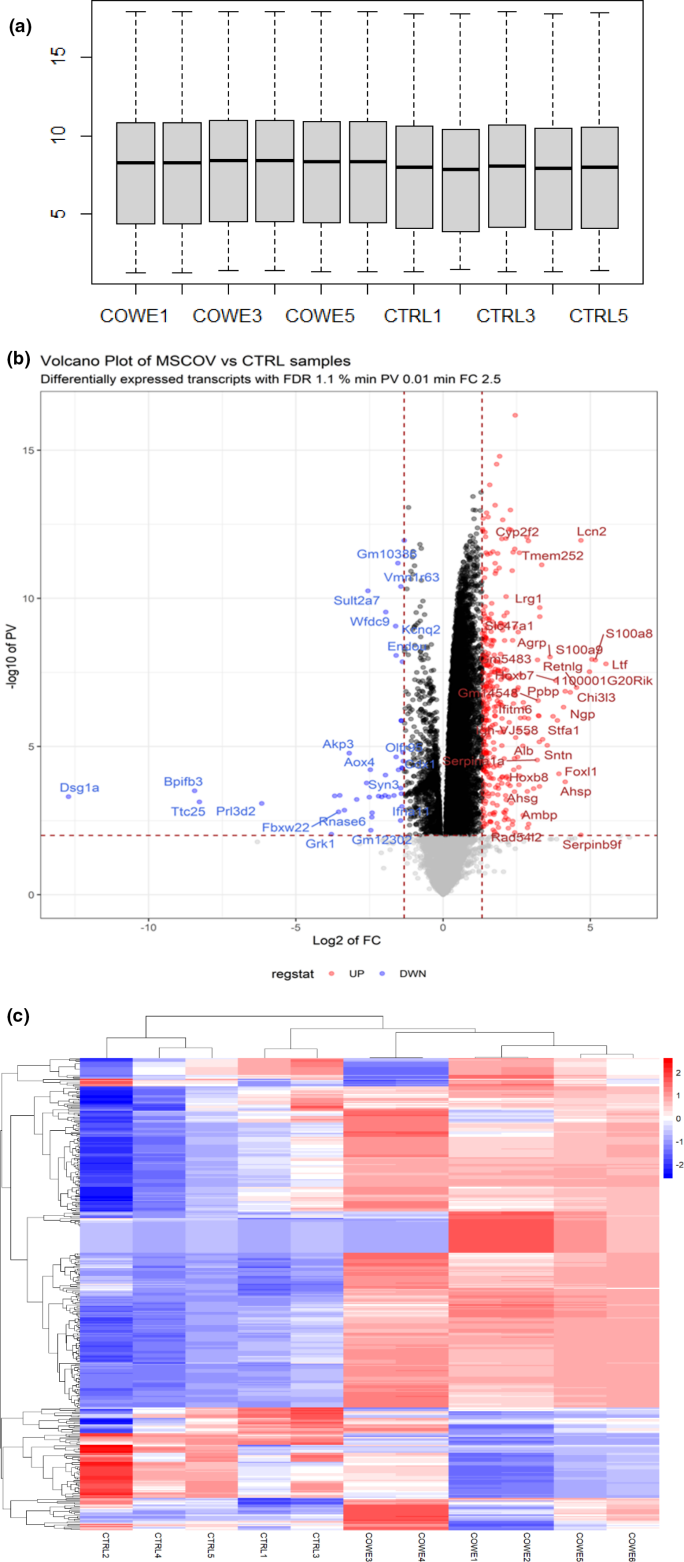
Microarray transcriptome analysis of control (*n* = 5) and *Capparis ovata* water extract (COWE‐treated) (*n* = 6) mice liver. (a) Box plot visualization of log2‐transformed normalized gene expression data. (b) Volcano plot depicting the number of differentially expressed genes (DEGs) between control and treatment. (c) Heat map of DEGs between groups (false discovery rate [FDR] = 0.01 and fold changes = 2.5).

In this study, we additionally utilized the whole‐genome transcriptome analysis to pick up knowledge into the molecular mechanisms that oversee the impact of *C. ovata* and to see how these procedures affect cell functions. A transcriptomic‐based screen of liver tissue taken from mice treated with COWE at 500 mg/kg/day for 28 days yielded 329 upregulated and 37 genes downregulated compared to control tissue (Figure 3).

**FIGURE 4:**
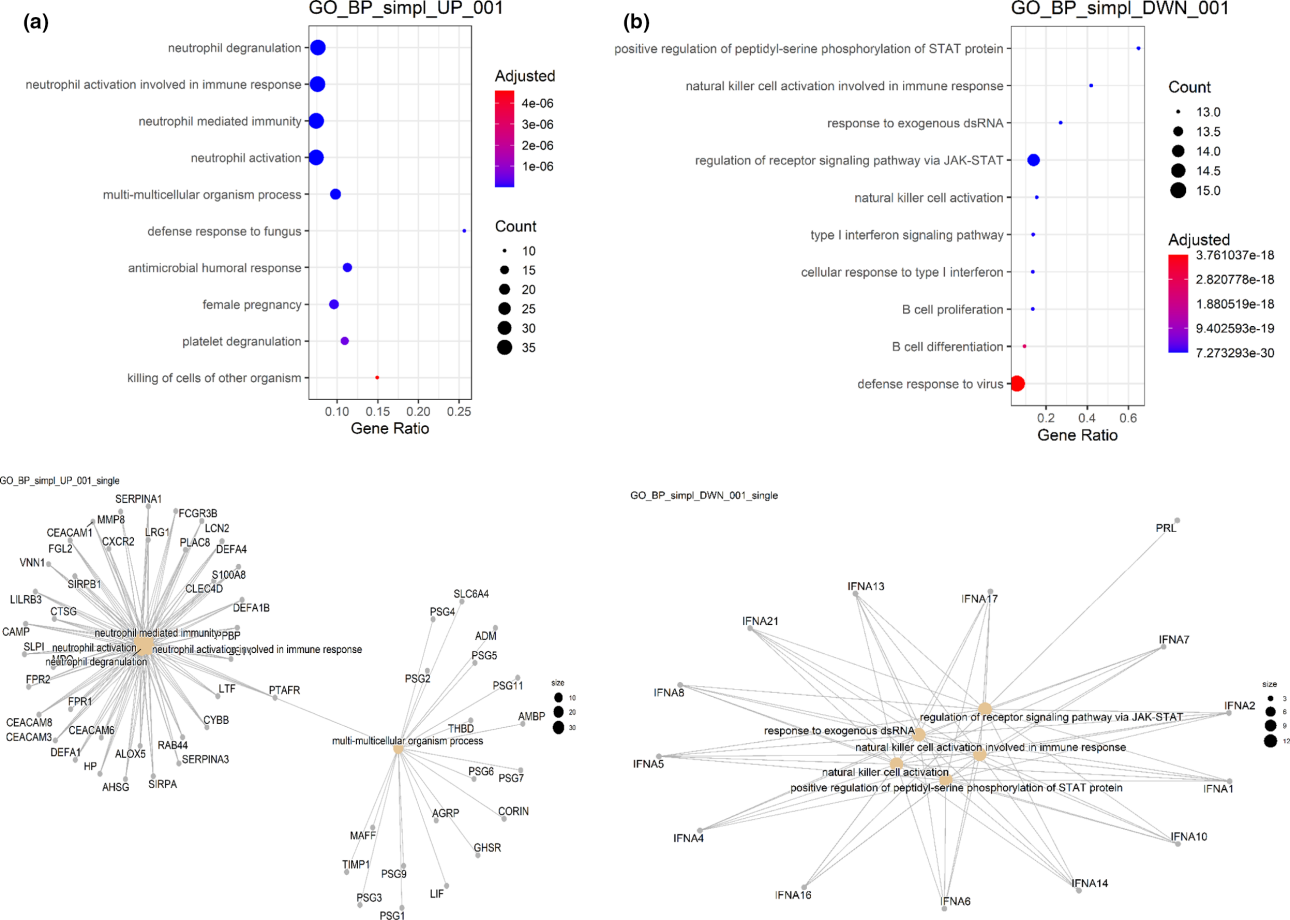
Gene ontology (GO) and Biological Process (BP) categories. (a) Overrepresented BP categories of upregulated genes and associated gene network. (b) Overrepresented BP categories of downregulated genes and associated gene network.

Utilizing these hits, we further performed Gene Ontology (GO) examination and recognized major modifications in GO classes for immune system processes and inflammatory responses. Neutrophil degranulation, neutrophil activation in immune responses, antimicrobial humoral response, defense response to fungus, response to LPS, and humoral immune response that were among upregulated genes and NK cell activation, cellular response to type I interferon, B‐cell proliferation and differentiation and regulation of JAK–STAT pathway that were in downregulated genes (Figure 4a,b). Most of the upregulated genes among the immune system and inflammatory response are related to the enhancing native immune system while negatively regulating the cellular immune responses and inflammation. These results, along with the changes previously reported (Ozgun‐Acar et al., 2016), highlight the *C. ovata* as a regulator of the transcripts in signaling immune response pathways. To further analyze these findings, we performed a gene network analysis of the up‐and downregulated genes (Figure 4a,b) and observed interactions among these genes. It implies that these genes have a more significant number of interactions among themselves. The examination of these functions suggested that the possible molecular basis of COWE exposure at the mentioned dose modulates the immune system at the molecular level.

### 3.3 Toxicological properties

**FIGURE 5:**
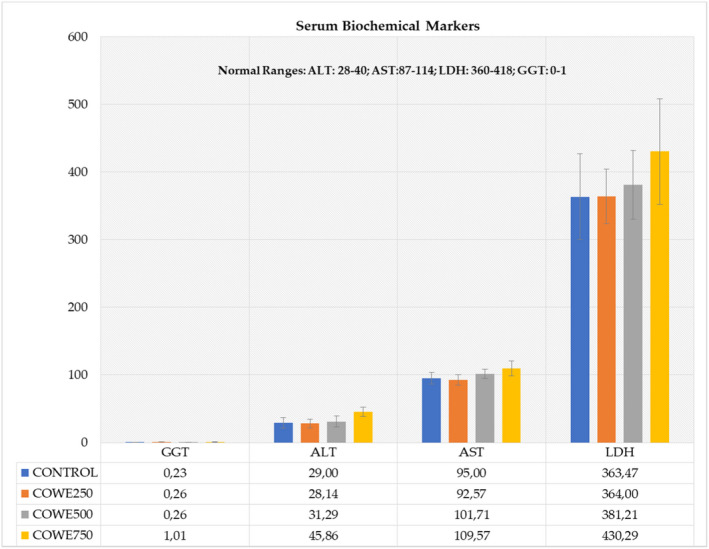
The effect of *Capparis ovata* water extract (COWE) (250, 500, and 750 mg/kg/day) treatments on serum alanine transaminase (ALT), aspartate transaminase (AST), and lactate dehydrogenase (LDH) enzyme activities in C57BL6 mice. All activities are expressed as IU/L (international units of enzyme per liter). The results are the averages of three independent set of experiments with duplicate measurements. Error bars represent standard deviations.

The further goal of this investigation was to see if COWE had any adverse effects on toxicity markers or outcomes in an organism. For this reason, first, ALT, AST, LDH, and GGT activities were determined as selected biochemical markers for liver damage as these enzymes are released during injury to liver cells or bile ducts as well as some other tissues and can be significantly elevated during tissue injuries (Giannini et al., 2005). None of these biomarker enzyme activities was found to be altered with 250 and 500 mg/kg/day COWE treatment in mice (Figure 5). All activities are considered to be in the normal range and not different from the values seen in control animals. However, very slight increases were noted with 750 mg/kg/day COWE treatment at ALT and LDH activities while the AST and GGT activities remained in the normal range. Therefore, unaltered biochemical markers of liver damage with 250–500 mg/kg/day COWE might be accepted as seemingly healthy doses.

The expression of 340 essential genes along housekeeping genes (B2M, ACTB, GAPDH) in 13 separate biological pathways triggered in response to xenobiotics was also profiled in this study applying the Molecular Toxicology Pathway Finder RT^2^ Profiler PCR array (Table S2). The pathways contained primers for oxidative stress & antioxidant responses, heat shock response, endoplasmic reticulum (ER) stress & unfolded protein responses, cytochrome P450 & phase 1 drug metabolism, steatosis, cholestasis, phospholipidosis, and immunotoxicity. The majority of the toxicological pathways represented by this array are interconnected, whereas the rest are autonomous. Inhibition of β‐oxidation, for example, causes steatosis, while uncoupling mitochondrial energy metabolism causes apoptosis and necrosis. This array can efficiently and accurately examine the expression of a specific panel of genes using real‐time PCR to determine which molecular toxicological responses are activated by the substances present in COWE. Three genes were discovered to be significantly altered (2.5‐fold, *p* ≤ .05) with 500 mg/kg/day COWE treatment, but no significant changes were found at 250 mg/kg/day COWE treatment. After 750 mg/kg/day of COWE treatment, 14 genes were downregulated, and 54 were upregulated (Table S3). At 500 mg/kg/day COWE, abnormal gene expression at DNA damage and repair (O^6^‐methylguanine‐DNA methyltransferase (MGMT)), heat shock protein (heat shock protein family B (small) member 7 (HSPB7)), and immunotoxicity (APOF) were seen, according to our biological pathway analysis. These pathways were little impacted since only a few genes were altered across them, and they were unrelated to one another (Table S2).

**FIGURE 6:**
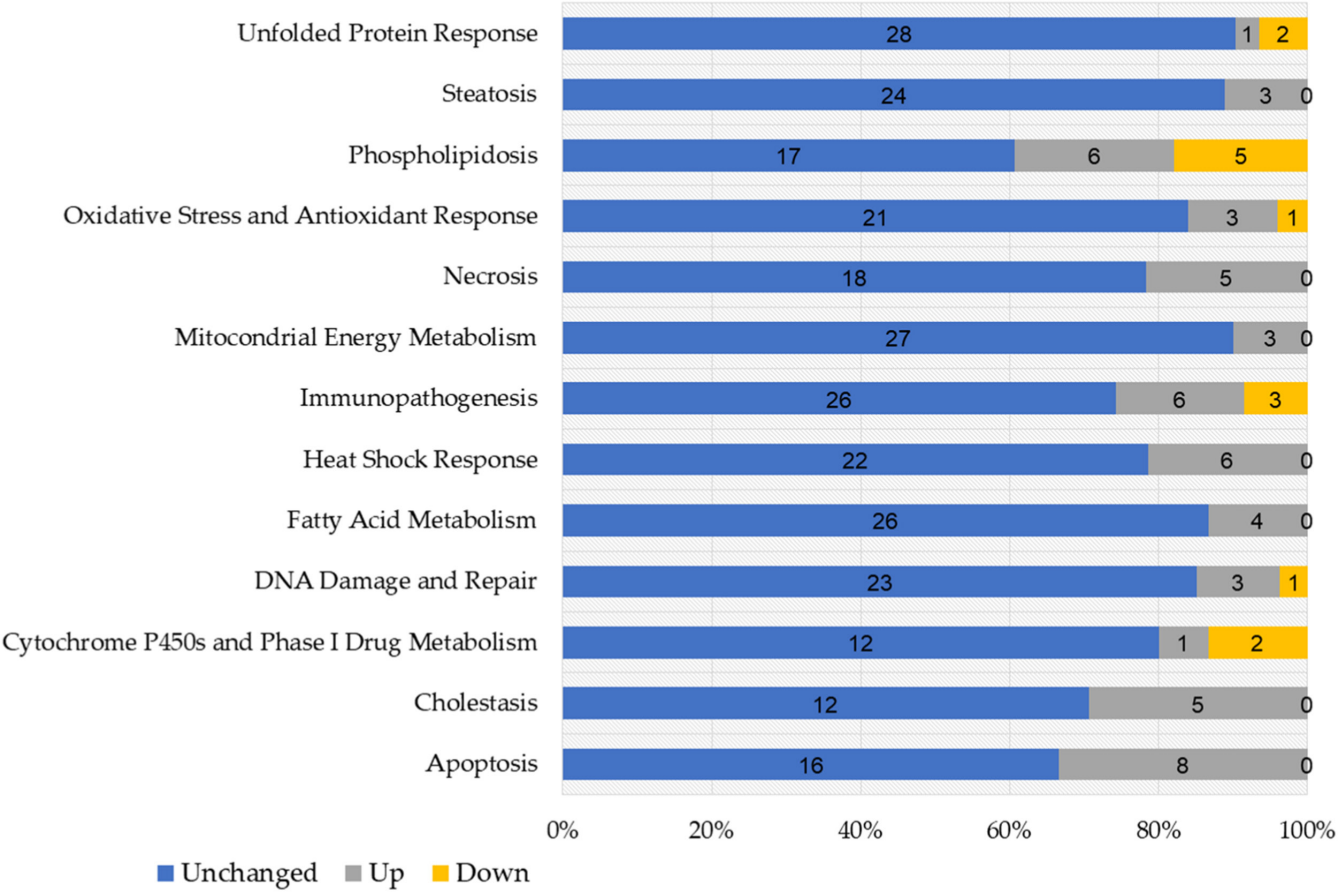
The number of significant gene expression changes generated by *Capparis ovata* water extract (COWE) (750 mg/kg) after 28 days of exposure was used to identify potential adverse effects. The numbers on the bars show gene expression changes in mouse liver from the Molecular Toxicology Pathway Finder RT^2^ PCR array that are unchanged, significantly up‐ and downregulated, respectively.

The direct and multifactorial effects of COWE (at 750 mg/kg/day) exposure were indicated by genes suggestive of many possible liver toxicities. COWE significantly affected up to 20% of the 340 genes on the Molecular Toxicology array at that dose (Figure 6 and Table S2). COWE changed the expression of genes linked to cholestasis, steatosis, and phospholipidosis, as well as genes related to the underlying mechanisms, such as altered fatty acid metabolism, oxidative stress, changes in mitochondrial energy, ER stress, and heat shock response, all of which can affect genes linked to DNA damage and repair, apoptosis, necrosis, inflammation, and immune response. The cholestasis, steatosis, and phospholipidosis categories exhibited the highest percentage of significant gene expression changes; however, it is unclear whether the transporter genes or receptors stand out or are a clear indication of cholestasis, steatosis, and phospholipidosis. CYP7A1, a gene involved in the conversion of cholesterol to bile acids, was downregulated. Several transporter genes and elimination routes may be disrupted, resulting in cholestatic hepatotoxicity (Nguyen et al., 2014). Furthermore, COWE alters several irregularly linked genes to fatty acid metabolism, implying that steatosis and phospholipidosis are less likely to develop. Hepatotoxicity‐related liver lipid deposits can also be ascribed to suppressing the electron chain’s respiratory complexes in the mitochondria, decreased energy (adenosine triphosphate) production, and oxidative stress (Boelsterli & Lim, 2007). This may cause apoptosis and necrosis (Jaeschke et al., 2002). COWE increased the expression of several apoptosis‐related genes as well as genes involved in DNA damage and repair.

Drugs and other compounds are oxidatively metabolized into more water‐soluble metabolites by cytochrome P450 (CYP450)‐dependent enzymes. Only three genes were likely regulated by high dose COWE treatment in the CYP450 and phase I drug metabolism. We found that CYP7A1 and FMO4 were downregulated and CYP1A2 upregulated with 750 mg/kg/day COWE treatment. Because CYP1A2 processes few drugs, the modest (2.73‐fold) upregulation of CYP1A2 may not be regarded as unfavorable drug–diet interactions, although caution should be exercised with coffee use (Tornio & Backman, 2018).

## 4 CONCLUSION

These findings clearly imply that COWE is an excellent source of nutrients, including proteins, polyunsaturated fatty acids, and minerals. The high total phenolic and flavonoid content, as well as the radical‐scavenging effect, demonstrates that it is a potent source of natural antioxidants. Furthermore, due to its potent anti‐inflammatory effects, it might be utilized as a supplementary treatment for various inflammatory illnesses such as multiple sclerosis and ulcerative colitis. And also, 250 and 500 mg/kg/day doses of *C. ovata* can be considered safe, while dangerous drug‐ and diet‐ interactions should be considered at or above 750 mg/kg/day doses.

## AUTHOR CONTRIBUTIONS

Alaattin Sen conceptualized, supervised, and managed the project, managed the data and acquired the funds, wrote the initial draft, and reviewed the manuscript; Ozden Ozgun‐Acar performed pharmacological analysis, Gurbet Celik‐Turgut did the toxicological experiment, Serdar Sezer handled biochemical analysis, and Huseyin Guner did statistical analysis; all authors reviewed and approved the final manuscript as submitted.

## CONFLICT OF INTEREST

The authors declared no potential conflicts of interest with respect to the research, authorship, and publication of this article.

## ETHICAL STATEMENT

Ethics approval was not required for this research.

## Supporting information


Table S1
Table S2.
Table S3.

## Data Availability

Data sharing not applicable to this article as no datasets were generated or analysed during the current study. and the data that support the findings of this study are available from the corresponding author upon reasonable request.
