# Supplementary material for: Biochemical, pharmacological, and toxicological attributes of caper (Capparis ovata) flowering buds and berries pickles
Source: Food Sci Nutr. 2022 Jul 30;10(12):4189–200. doi: 10.1002/fsn3.3012 (PMC9731540; doi:10.1002/fsn3.3012)
Supplement: Supplementary file 1 — Table S1 Table S2. Table S3. [file FSN3-10-4189-s001.docx]

**SUPPLEMENTARY MATERIAL**

**Biochemical, Pharmacological and Toxicological Attributes of Caper (*Capparis ovata*) Flowering Buds and Berries Pickles**

Ozden OZGUN-ACAR^1^, Gurbet CELIK-TURGUT^2^, Hüseyin GUNER^3^, Serdar SEZER^4,5^ and Alaattin SEN^3,6*^

*^1^Pamukkale University, Seed Breeding & Genetics Application Research Center, 20070 Denizli, Turkey*

*^2^Pamukkale University, Faculty of Applied Sciences, Organic Agriculture Management, Civril, 20680 Denizli, Turkey*

*^3^Abdullah Gul University, Faculty of Life and Natural Sciences, Department of Molecular Biology and Genetics, 38080 Kayseri, Turkey*

*^4^TUBITAK, Institute of Chemical Technology, Marmara Research Center, 41470 Kocaeli, Turkey*

*^5^Suleyman Demirel University, Faculty of Medicine, Department of Pharmacology, Medicine, Medical Devices and Dermocosmetic Research and Application Laboratory - IDAL, 32260 Isparta, Turkey*

*^6^Pamukkale University Department of Biology, Faculty of Arts & Sciences, 20070 Denizli, Turkey*

**Table S1.** Molecular Toxicology Pathway Finder RT2 Profiler PCR array gene list

| **Gene** | **Description** |
| --- | --- |
| **Apoptosis** | |
| AbI1 | C-abl oncogene 1, non-receptor tyrosine kinase |
| Akt1 | V-akt murine thymoma viral oncogene homolog 1 |
| Apaf1 | Apoptotic peptidase activating factor 1 |
| Bad | BCL2-associated agonist of cell death |
| Bak1 | BCL2-antagonist/killer 1 |
| Bcl2I11 | BCL2-like 11 (apoptosis facilitator) |
| Bid | BH3 interacting domain death agonist |
| Birc3 | Baculoviral IAP repeat containing 3 |
| Casp1 | Caspase 1, apoptosis-related cysteine peptidase (interleukin 1, beta, convertase) |
| Casp2 | Caspase 2, apoptosis-related cysteine peptidase |
| Casp3 | Caspase 3, apoptosis-related cysteine peptidase |
| Casp7 | Caspase 7, apoptosis-related cysteine peptidase |
| Casp8 | Caspase 8, apoptosis-related cysteine peptidase |
| Casp9 | Caspase 9, apoptosis-related cysteine peptidase |
| Cd40Ig | CD40 ligand |
| Cflar | CASP8 and FADD-like apoptosis regulator |
| Fadd | Fas (TNFRSF6)-associated via death domain |
| Fas | Fas (TNF receptor superfamily, member 6) |
| Gadd45a | Growth arrest and DNA-damage-inducible, alpha |
| McI1 | Myeloid cell leukemia sequence 1 (BCL2-related) |
| TNfrsf10 | Tumor necrosis factor (ligand) superfamily, member 10 |
| Tnfrsf10b | Tumor necrosis factor receptor superfamily, member 10b |
| Trp53 | Tumor protein p53 |
| Xiap | X-linked inhibitor of apoptosis |
| **Necrosis** | |
| Atp6v1g2 | ATPase, H+ transporting, lysosomal 13kDa, V1 subunit G2 |
| G10rik | Riken cdna 9430015g10 gene |
| Bmf | Bcl2 modifying factor |
| Commd4 | COMM domain containing 4 |
| Cyld | Cylindromatosis (turban tumor syndrome) |
| Defb1 | Defensin, beta 1 |
| Dpysl4 | Dihydropyrimidinase-like 4 |
| Eif5b | Eukaryotic translation initiation factor 5B |
| Foxi1 | Forkhead box I1 |
| Galnt5 | UDP-N-acetyl-alpha-D-galactosamine: polypeptideN-acetylgalactosaminyltransferase 5 (GalNAc-T5) |
| Hspbap1 | HSPB (heat shock 27kDa) associated protein 1 |
| Jph3 | Junctophilin 3 |
| Kcnip1 | Kv channel interacting protein 1 |
| Nudt13 | Nudix (nucleoside diphosphate linked moiety X)-type motif 13 |
| Parp2 | Poly (ADP-ribose) polymerase 2 |
| Pvr | Poliovirus receptor |
| Rab25 | RAB25, member RAS oncogene family |
| S100a7a | S100 calcium binding protein A7A |
| Spata2 | Spermatogenesis associated 2 |
| Sycp2 | Synaptonemal complex protein 2 |
| Tmem57 | Transmembrane protein 57 |
| Tnfaip8l1 | Tumor necrosis factor, alpha-induced protein 8-like 1 |
| Txnl4b | Thioredoxin-like 4B |
| **DNA Damage and Repair** | |
| Apex1 | APEX nuclease (multifunctional DNA repair enzyme) 1 |
| Atm | Ataxia telangiectasia mutated |
| Brca1 | Breast cancer 1, early onset |
| Brca2 | Breast cancer 2, early onset |
| Cdkn1a | Cyclin-dependent kinase inhibitor 1A (p21, Cip1) |
| Chek1 | CHK1 checkpoint homolog (S. pombe) |
| Chek2 | CHK2 checkpoint homolog (S. pombe) |
| Ddit3 | DNA-damage-inducible transcript 3 |
| Ercc1 | Excision repair cross-complementing rodent repair deficiency, complementation group 1 (includes overlapping antisense sequence) |
| Ercc2 | Excision repair cross-complementing rodent repair deficiency, complementation group 2 |
| Ercc3 | Excision repair cross-complementing rodent repair deficiency, complementation group 3 (xeroderma pigmentosum group B complementing) |
| Ercc5 | Excision repair cross-complementing rodent repair deficiency, complementation group 5 |
| Ercc6 | Excision repair cross-complementing rodent repair deficiency, complementation group 6 |
| Lig4 | Ligase IV, DNA, ATP-dependent |
| Mdm2 | Mdm2 p53 binding protein homolog (mouse) |
| Mgmt | O-6-methylguanine-DNA methyltransferase |
| MIh1 | MutL homolog 1, colon cancer, nonpolyposis type 2 (E. coli) |
| Msh1 | MutL homolog 1, colon cancer, nonpolyposis type 2 (E. coli) |
| Ogg1 | 8-oxoguanine DNA glycosylase |
| Parp1 | Poly (ADP-ribose) polymerase 1 |
| Pcna | Proliferating cell nuclear antigen |
| Prkdc | Protein kinase, DNA-activated, catalytic polypeptide |
| Rad51 | RAD51 homolog (S. cerevisiae) |
| Xpa | Xeroderma pigmentosum, complementation group A |
| Xpc | Xeroderma pigmentosum, complementation group C |
| Xrcc1 | X-ray repair complementing defective repair in Chinese hamster cells 1 |
| Xrcc5 | X-ray repair complementing defective repair in Chinese hamster cells 5 (double-strand-break rejoining) |
| **Mitocondrial Energy Metabolism** | |
| Acly | ATP citrate lyase |
| Aco1 | Aconitase 1, soluble |
| Aco2 | Aconitase 2, mitochondrial |
| Cox6b1 | Cytochrome c oxidase subunit Vib polypeptide 1 (ubiquitous) |
| Cox8a | Cytochrome c oxidase subunit VIIIA (ubiquitous) |
| Cs | Citrate synthase |
| Cyc1 | Cytochrome c-1 |
| Dlb | Dihydrolipoamide dehydrogenase |
| Dlst | Dihydrolipoamide S-succinyltransferase (E2 component of 2-oxo-glutarate complex) |
| Fh1 | Fumarate hydratase |
| Idh1 | Isocitrate dehydrogenase 1 (NADP+), soluble |
| Cox7a2 | Cytochrome c oxidase subunit VIIa polypeptide 2 |
| Idh2 | Isocitrate dehydrogenase 2 (NADP+), mitochondrial |
| Idh3a | Isocitrate dehydrogenase 3 (NAD+) alpha |
| Idh3b | Isocitrate dehydrogenase 3 (NAD+) beta |
| Idh3g | Isocitrate dehydrogenase 3 (NAD+) gamma |
| Mdh1 | Malate dehydrogenase 1, NAD (soluble) |
| Mdh1b | Malate dehydrogenase 1B, NAD (soluble) |
| Mdh2 | Malate dehydrogenase 2, NAD (mitochondrial) |
| Ogdh1 | Oxoglutarate (alpha-ketoglutarate) dehydrogenase (lipoamide) |
| Sdha | Succinate dehydrogenase complex, subunit A, flavoprotein (Fp) |
| Sdhb | Succinate dehydrogenase complex, subunit B, iron sulfur (Ip) |
| Sdhc | Succinate dehydrogenase complex, subunit C, integral membrane protein, 15kDa |
| Sdhd | Succinate dehydrogenase complex, subunit D, integral membrane protein |
| Suclg1 | Succinate-CoA ligase, alpha subunit |
| Sucla2 | Succinate-CoA ligase, ADP-forming, beta subunit |
| Suclg2 | Succinate-CoA ligase, GDP-forming, beta subunit |
| Ucp1 | Uncoupling protein 1 (mitochondrial, proton carrier) |
| Ucp2 | Uncoupling protein 2 (mitochondrial, proton carrier) |
| Ucp3 | Uncoupling protein 3 (mitochondrial, proton carrier) |
| **Fatty Acid Metabolism** | |
| Acaa1 | Acetyl-CoA acyltransferase 1 |
| Acaa2 | Acetyl-CoA acyltransferase 2 |
| Acadl | Acyl-CoA dehydrogenase, long chain |
| Acad11 | Acyl-CoA dehydrogenase family, member 11 |
| Acad9 | Acyl-CoA dehydrogenase family, member 9 |
| Acadm | Acyl-CoA dehydrogenase, C-4 to C-12 straight chain |
| Acads | Acyl-CoA dehydrogenase, C-2 to C-3 short chain |
| Acadsb | Acyl-CoA dehydrogenase, short/branched chain |
| AcadvI | Acyl-CoA dehydrogenase, very long chain |
| Acat1 | Acetyl-CoA acetyltransferase 1 |
| Acat2 | Acetyl-CoA acetyltransferase 2 |
| Acot12 | Acyl-CoA thioesterase 12 |
| Acot2 | Acyl-CoA thioesterase 2 |
| Acot6 | Acyl-CoA thioesterase 6 |
| Acot7 | Acyl-CoA thioesterase 7 |
| Acot8 | Acyl-CoA thioesterase 8 |
| Acot9 | Acyl-CoA thioesterase 9 |
| Acox1 | Acyl-CoA oxidase 1, palmitoyl |
| Acox2 | Acyl-CoA oxidase 2, branched chain |
| Acox3 | Acyl-CoA oxidase 3, pristanoyl |
| Cpt1a | Carnitine palmitoyltransferase 1A (liver) |
| Cpt1b | Carnitine palmitoyltransferase 1B (muscle) |
| Cpt2 | Carnitine palmitoyltransferase 2 |
| Crat | Carnitine O-acetyltransferase |
| Crot | Carnitine O-octanoyltransferase |
| Echs1 | Enoyl CoA hydratase, short chain, 1, mitochondrial |
| Ehhadh | Enoyl-CoA, hydratase/3-hydroxyacyl CoA dehydrogenase |
| Gcdh | Glutaryl-CoA dehydrogenase |
| Hadha | Hydroxyacyl-CoA dehydrogenase/3-ketoacyl-CoA thiolase/enoyl-CoA hydratase (trifunctional protein), alpha subunit |
| **Oxidative Stress and Antioxidant Response** | |
| Aass | Aminoadipate-semialdehyde synthase |
| Cat | Catalase |
| Ctsb | Cathepsin B |
| Dhcr24 | 24-dehydrocholesterol reductase |
| Duox1 | Dual oxidase 1 |
| Duox2 | Dual oxidase 2 |
| Epx | Eosinophil peroxidase |
| Gpx2 | Glutathione peroxidase 2 (gastrointestinal) |
| Gpx3 | Glutathione peroxidase 3 (plasma) |
| Gpx4 | Glutathione peroxidase 4 (phospholipid hydroperoxidase) |
| Gpx5 | Glutathione peroxidase 5 (epididymal androgen-related protein) |
| Gpx6 | Glutathione peroxidase 6 (olfactory) |
| Gpx7 | Glutathione peroxidase 7 |
| Mpo | Myeloperoxidase |
| Nqo1 | NAD(P)H dehydrogenase, quinone 1 |
| Nudt1 | Nudix (nucleoside diphosphate linked moiety X)-type motif 1 |
| Nudt15 | Nudix (nucleoside diphosphate linked moiety X)-type motif 15 |
| Ppp1r15b | Protein phosphatase 1, regulatory (inhibitor) subunit 15B |
| Prdx1 | Peroxiredoxin 1 |
| Prdx2 | Peroxiredoxin 2 |
| Prdx6 | Peroxiredoxin 6 |
| Tpo | Thyroid peroxidase |
| Txnip | Thioredoxin interacting protein |
| Txnrd2 | Thioredoxin reductase 2 |
| Ucp3 | Uncoupling protein 3 (mitochondrial, proton carrier) |
| **Heat Shock Response** | |
| Cryaa | Crystallin, alpha A |
| Cryab | Crystallin, alpha B |
| Dnaja1 | DnaJ (Hsp40) homolog, subfamily A, member 1 |
| Dnaja2 | DnaJ (Hsp40) homolog, subfamily A, member 2 |
| Dnaja3 | DnaJ (Hsp40) homolog, subfamily A, member 3 |
| Dnajb1 | DnaJ (Hsp40) homolog, subfamily B, member 1 |
| Dnajb6 | DnaJ (Hsp40) homolog, subfamily B, member 6 |
| Dnajc3 | DnaJ (Hsp40) homolog, subfamily C, member 3 |
| Dnajc5 | DnaJ (Hsp40) homolog, subfamily C, member 5 |
| Dnajc6 | DnaJ (Hsp40) homolog, subfamily C, member 6 |
| Hsf1 | Heat shock transcription factor 1 |
| Hsf2 | Heat shock transcription factor 2 |
| Hsp90aa1 | Heat shock protein 90kDa alpha (cytosolic), class A member 1 |
| Hspa1a | Heat shock 70kDa protein 1A |
| Hspa1I | Heat shock 70kDa protein 1-like |
| Hspa2 | Heat shock 70kDa protein 2 |
| Hspa4 | Heat shock 70kDa protein 4 |
| Hspa5 | Heat shock 70kDa protein 5 (glucose-regulated protein, 78kDa) |
| Hspa8 | Heat shock 70kDa protein 8 |
| Hspa9 | Heat shock 70kDa protein 9 (mortalin) |
| Hspb1 | Heat shock 27kDa protein 1 |
| Hspb2 | Heat shock 27kDa protein 2 |
| Hspb6 | Heat shock protein, alpha-crystallin-related, B6 |
| Hspb8 | Heat shock 22kDa protein 8 |
| Hspd1 | Heat shock 60kDa protein 1 (chaperonin) |
| Hspe1 | Heat shock 10kDa protein 1 (chaperonin 10) |
| Hsph1 | Heat shock 105kDa/110kDa protein 1 |
| Tcp1 | T-complex 1 |
| **Unfolded Protein Response** | |
| Amfr | Autocrine motility factor receptor |
| Atf4 | Activating transcription factor 4 (tax-responsive enhancer element B67) |
| Atf6 | Activating transcription factor 6 |
| Ddit3 | DNA-damage-inducible transcript 3 |
| Derl1 | Der1-like domain family, member 1 |
| Edem1 | ER degradation enhancer, mannosidase alpha-like 1 |
| Edem3 | ER degradation enhancer, mannosidase alpha-like 3 |
| Eif2ak3 | Eukaryotic translation initiation factor 2-alpha kinase 3 |
| Ern2 | Endoplasmic reticulum to nucleus signaling 2 |
| Ero1l | ERO1-like (S. cerevisiae) |
| Ero1lb | ERO1-like beta (S. cerevisiae) |
| Fbxo6 | F-box protein 6 |
| Herpud1 | Homocysteine-inducible, endoplasmic reticulum stress-inducible, ubiquitin-like domain member 1 |
| Htra2 | HtrA serine peptidase 2 |
| Htra4 | HtrA serine peptidase 4 |
| Mbtps1 | Membrane-bound transcription factor peptidase, site 1 |
| Mbtps2 | Membrane-bound transcription factor peptidase, site 2 |
| Nploc4 | Nuclear protein localization 4 homolog (S. cerevisiae) |
| Nucb1 | Nucleobindin 1 |
| Os9 | Osteosarcoma amplified 9, endoplasmic reticulum lectin |
| Pfdn5 | Prefoldin subunit 5 |
| Ppia | Peptidylprolyl isomerase A (cyclophilin A) |
| Sec62 | SEC62 homolog (S. cerevisiae) |
| Sel1l | Sel-1 suppressor of lin-12-like (C. elegans) |
| Serp1 | Stress-associated endoplasmic reticulum protein 1 |
| Syvn1 | Synovial apoptosis inhibitor 1, synoviolin |
| Ube2g2 | Ubiquitin-conjugating enzyme E2G 2 |
| Ube2j2 | Ubiquitin-conjugating enzyme E2, J2 |
| Ubxn4 | UBX domain protein 4 |
| Vcp | Valosin containing protein |
| Xbp1 | X-box binding protein 1 |
| **Cytochrome P450s and Phase I Drug Metabolism** | |
| Cyp1a1 | Cytochrome P450, family 1, subfamily A, polypeptide 1 |
| Cyp1a2 | Cytochrome P450, family 1, subfamily A, polypeptide 2 |
| Cyp2d22 | Cytochrome P450, family 2, subfamily D, polypeptide 22 |
| Cyp2e1 | Cytochrome P450, family 2, subfamily E, polypeptide 1 |
| Cyp3a11 | Cytochrome P450, family 3, subfamily A, polypeptide 11 |
| Cyp4a14 | Cytochrome P450, family 4, subfamily A, polypeptide 14 |
| Cyp7a1 | Cytochrome P450, family 7, subfamily A, polypeptide 1 |
| Cyp7b1 | Cytochrome P450, family 7, subfamily B, polypeptide 1 |
| Esd | Esterase D |
| Fmo2 | Flavin containing monooxygenase 2 (non-functional) |
| Fmo3 | Flavin containing monooxygenase 3 |
| Fmo4 | Flavin containing monooxygenase 4 |
| Fmo5 | Flavin containing monooxygenase 5 |
| Maoa | Monoamine oxidase A |
| Maob | Monoamine oxidase B |
| **Steatosis** | |
| Acaca | Acetyl-CoA carboxylase alpha |
| Adk | Adenosine kinase |
| Aldh2 | Aldehyde dehydrogenase 2 family (mitochondrial) |
| Aqp4 | Aquaporin 4 |
| Cd36 | CD36 molecule (thrombospondin receptor) |
| Comt | Catechol-O-methyltransferase |
| Dnm1 | Dynamin 1 |
| Eno1 | Enolase 1, (alpha) |
| Fasn | Fatty acid synthase |
| Gpd1 | Glycerol-3-phosphate dehydrogenase 1 (soluble) |
| Haao | 3-hydroxyanthranilate 3,4-dioxygenase |
| Hadhb | Hydroxyacyl-CoA dehydrogenase/3-ketoacyl-CoA thiolase/enoyl-CoA hydratase (trifunctional protein), beta subunit |
| Khk | Ketohexokinase (fructokinase) |
| Lmna | Lamin A/C |
| Lpl | Lipoprotein lipase |
| Ly6d | Lymphocyte antigen 6 complex, locus D |
| Mapk8 | Mitogen-activated protein kinase 8 |
| Mttp | Microsomal triglyceride transfer protein |
| Pcca | Propionyl CoA carboxylase, alpha polypeptide |
| Pnpla3 | Patatin-like phospholipase domain containing 3 |
| Ppara | Peroxisome proliferator-activated receptor alpha |
| Pparg | Peroxisome proliferator-activated receptor gamma |
| Retn | Resistin |
| Scd1 | Stearoyl-CoA desaturase (delta-9-desaturase) |
| Srebf1 | Sterol regulatory element binding transcription factor 1 |
| Syt1 | Synaptotagmin I |
| Tff3 | Trefoil factor 3 (intestinal) |
| **Cholestasis** | |
| Abcb1a | ATP-binding cassette, sub-family B (MDR/TAP), member 1 |
| Abcb4 | ATP-binding cassette, sub-family B (MDR/TAP), member 4 |
| Abcc1 | ATP-binding cassette, sub-family C (CFTR/MRP), member 1 |
| Abcc2 | ATP-binding cassette, sub-family C (CFTR/MRP), member 2 |
| Abcc3 | ATP-binding cassette, sub-family C (CFTR/MRP), member 3 |
| Apoe | Apolipoprotein E |
| Atp8b1 | ATPase, aminophospholipid transporter, class I, type 8B, member 1 |
| Dlat | Dihydrolipoamide S-acetyltransferase |
| Esr1 | Estrogen receptor 1 |
| Jag1 | Jagged 1 |
| Nr1h4 | Nuclear receptor subfamily 1, group H, member 4 |
| Nup210 | Nucleoporin 210kDa |
| Osta | Organic solute transporter alpha |
| Ostb | Organic solute transporter beta |
| Pdyn | Prodynorphin |
| Rdx | Radixin |
| Slc10a1 | Solute carrier family 10 (sodium/bile acid cotransporter family), member 1 |
| **Phospholipidosis** | |
| Abcb1b | ATP-binding cassette, sub-family B (MDR/TAP), member 1B |
| Aldh1a1 | Aldehyde dehydrogenase 1 family, member A1 |
| Asah1 | N-acylsphingosine amidohydrolase (acid ceramidase) 1 |
| Asns | Asparagine synthetase (glutamine-hydrolyzing) |
| Ces2c | Carboxylesterase 2 |
| Ephx1 | Epoxide hydrolase 1, microsomal (xenobiotic) |
| Fabp1 | Fatty acid binding protein 1, liver |
| Fxc1 | Fracture callus 1 homolog (rat) |
| Gstm4 | Glutathione S-transferase mu 4 |
| Hpn | Hepsin |
| Inhbe | inhibin, beta E |
| Lss | Lanosterol synthase (2,3-oxidosqualene-lanosterol cyclase) |
| Manba | Mannosidase, beta A, lysosomal |
| Mlx | MAX-like protein X |
| Mrps18b | Mitochondrial ribosomal protein S18B |
| Nr0b2 | Nuclear receptor subfamily 0, group B, member 2 |
| Por | P450 (cytochrome) oxidoreductase |
| S100a8 | S100 calcium binding protein A8 |
| Sc4mol | Sterol-C4-methyl oxidase-like |
| Serpina3n | Serpin peptidase inhibitor, clade A (alpha-1 antiproteinase, antitrypsin), member 3 |
| Slco1a2 | Solute carrier organic anion transporter family, member 1A2 |
| Smpd1 | Sphingomyelin phosphodiesterase 1, acid lysosomal |
| Stbd1 | Starch binding domain 1 |
| Tagln | Transgelin |
| Ugt1a1 | UDP glucuronosyltransferase 1 family, polypeptide A1 |
| Ugt2a1 | UDP glucuronosyltransferase 2 family, polypeptide A1, complex locus |
| Ugt2b1 | UDP glucuronosyltransferase 2 family, polypeptide B1 |
| Wipi1 | WD repeat domain, phosphoinositide interacting 1 |
| **Immunopathogenesis** |  |
| Adh1 | Alcohol dehydrogenase 1C (class I), gamma polypeptide |
| Ahr | Aryl hydrocarbon receptor |
| Ahsg | Alpha-2-HS-glycoprotein |
| Alb | Albumin |
| Apoa5 | Apolipoprotein A-V |
| Apof | Apolipoprotein F |
| C3 | Complement component 3 |
| C9 | Complement component 9 |
| Cd19 | CD19 molecule |
| Cd80 | CD80 molecule |
| Cd86 | CD86 molecule |
| Cd8a | CD8a molecule |
| Ctse | Cathepsin E |
| Ep300 | E1A binding protein p300 |
| F2 | Coagulation factor II (thrombin) |
| Gpt | Glutamic-pyruvate transaminase (alanine aminotransferase) |
| Gsta3 | Glutathione S-transferase alpha 3 |
| Hpx | Hemopexin |
| Hrg | Histidine-rich glycoprotein |
| II1a | Interleukin 1, alpha |
| II2 | Interleukin 2 |
| IL4 | Interleukin 4 |
| IL5 | Interleukin 5 (colony-stimulating factor, eosinophil) |
| IL6 | Interleukin 6 (interferon, beta 2) |
| Itgax | Integrin, alpha X (complement component 3 receptor 4 subunit) |
| Klf1 | Kruppel-like factor 1 (erythroid) |
| Lyz2 | Lysozyme |
| Metap2 | Methionyl aminopeptidase 2 |
| Mki67 | Antigen identified by monoclonal antibody Ki-67 |
| Nr5a2 | Nuclear receptor subfamily 5, group A, member 2 |
| Pon1 | Paraoxonase 1 |
| Pou3f3 | POU class 3 homeobox 3 |
| Ptgs2 | Prostaglandin-endoperoxide synthase 2 (prostaglandin G/H synthase and cyclooxygenase) |
| Ptprc | Protein tyrosine phosphatase, receptor type, C |
| Trim10 | Tripartite motif containing 10 |
| Ubqln2 | Ubiquilin 2 |

**Table S2.** The genes whose expression levels were altered by the COWE (500 and 750 mg/kg/day) treatments in C57BL6 mice liver as compared to the untreated control animal. The results are the averages of three independent set of experiments with triplicate measurements.

| **Gene** | **Increase/Decrease (fold)** | | **Pathway Involved** |
| --- | --- | --- | --- |
|  | **500mg/kg/day** | **750mg/kg/day** |  |
| APAF1 |  | 2.74 | Apoptosis |
| BAD |  | 3.99 | Apoptosis |
| BAK1 |  | 2.71 | Apoptosis |
| BCL2T11 |  | 4.07 | Apoptosis |
| BIRC3 |  | 2.53 | Apoptosis |
| CD40LG |  | 2.98 | Apoptosis |
| CFLAR |  | 2.60 | Apoptosis |
| FAS |  | 5.12 | Apoptosis |
| ATP6V1G2 |  | 3.16 | Necrosis |
| G10RIK |  | 2.62 | Necrosis |
| GALNT5 |  | 3.44 | Necrosis |
| HSPBAP1 |  | 2.56 | Necrosis |
| TXNL4B |  | 2.85 | Necrosis |
| MGMT | 2.74 | 3.23 | DNA damage and repair |
| OGG1 |  | 3.78 | DNA damage and repair |
| XPC |  | 2.99 | DNA damage and repair |
| ATM |  | -2.66 | DNA damage and repair |
| MDH1 |  | 2.98 | Mitochondrial energy metabolism |
| UCP1 |  | 3.41 | Mitochondrial energy metabolism |
| UCP3 |  | 3.47 | Mitochondrial energy metabolism |
| ACAT2 |  | 3.11 | Fatty acid metabolism |
| ACOT2 |  | 3.03 | Fatty acid metabolism |
| ACOT3 |  | 4.54 | Fatty acid metabolism |
| EHHADH |  | 3.67 | Fatty acid metabolism |
| DUOX2 |  | 3.41 | Oxidative Stress and Antioxidant Response |
| GPX5 |  | 2.63 | Oxidative Stress and Antioxidant Response |
| UCP3 |  | 3.47 | Oxidative Stress and Antioxidant Response |
| GPX6 |  | -2.69 | Oxidative Stress and Antioxidant Response |
| CRYAA |  | 2.72 | Heat shock response |
| HSP90AA1 |  | 4.32 | Heat shock response |
| HSPA8 |  | 2.85 | Heat shock response |
| HSPB1 |  | 4.00 | Heat shock response |
| HSPB7 | 2.61 | 3.17 | Heat shock response |
| HSPE1 |  | 4.66 | Heat shock response |
| ERO1LB |  | -2.53 | Unfolded protein response |
| HERPUD1 |  | -2.70 | Unfolded protein response |
| PPIA |  | 4.71 | Unfolded protein response |
| CYP1A2 |  | 2.73 | Drug metabolism |
| CYP4A14 |  | -3.34 | Drug metabolism |
| FMO3 |  | -2.80 | Drug metabolism |
| COMT |  | 3.19 | Steatosis |
| PNPLA3 |  | 4.27 | Steatosis |
| PPARA |  | 7.66 | Steatosis |
| ABCC3 |  | 3.15 | Cholestasis |
| APOE |  | 2.97 | Cholestasis |
| ESR1 |  | 2.64 | Cholestasis |
| OSTB |  | 4.69 | Cholestasis |
| PDYN |  | 4.28 | Cholestasis |
| ABCB1B |  | -2.91 | Phospholipidosis |
| ALDH1A1 |  | 4.29 | Phospholipidosis |
| CES2C |  | 4.01 | Phospholipidosis |
| EPHX1 |  | 3.37 | Phospholipidosis |
| FABP1 |  | 6.90 | Phospholipidosis |
| HPN |  | -2.97 | Phospholipidosis |
| NROB2 |  | 2.84 | Phospholipidosis |
| S100A8 |  | -3.73 | Phospholipidosis |
| SERPINA3N |  | -3.39 | Phospholipidosis |
| SMPD1 |  | 5.74 | Phospholipidosis |
| WIPI1 |  | -4.99 | Phospholipidosis |
| ADH1 |  | -3.81 | Immunotoxicity |
| AHR |  | 2.59 | Immunotoxicity |
| APOF | 2.77 | 2.90 | Immunotoxicity |
| C3 |  | 2.68 | Immunotoxicity |
| CD8A |  | -5.00 | Immunotoxicity |
| HPX |  | 3.57 | Immunotoxicity |
| HRG |  | 2.94 | Immunotoxicity |
| IL5 |  | 4.60 | Immunotoxicity |
| UOX |  | -4.15 | Immunotoxicity |

(- : decrease)

**Table S3.** Residual pesticide concentrations in COWE.

| **Pesticide** | **MRL (mg/kg)** | **Conc. (mg/kg)** | **Pesticide** | **MRL (mg/kg)** | **Conc. (mg/kg)** |
| --- | --- | --- | --- | --- | --- |
| 2,4-Dimethylaniline | < 0.01 | N.D. | Hexaconazole | < 0.01 | N.D. |
| 2,4-Dimethylformamide | < 0.01 | N.D. | Hexythiazox | < 0.01 | N.D. |
| 2-methyl-4-chlorophenoxyacetic acid | < 0.01 | N.D. | Imazalil | < 0.01 | N.D. |
| 2.4.5-Trichlorophenoxyacetic acid | < 0.01 | N.D. | Imazamox | < 0.01 | N.D. |
| Acephate | < 0.01 | N.D. | Imidachloprid | < 0.01 | N.D. |
| Acetamipride | < 0.01 | N.D. | Indoxacarb | < 0.01 | N.D. |
| Aclonifen | < 0.01 | N.D. | Ioxynil | < 0.01 | N.D. |
| Alachlor | < 0.01 | N.D. | Iprodione | < 0.01 | N.D. |
| Atrazine | < 0.01 | N.D. | Iprovicarb | < 0.01 | N.D. |
| Benalaxyl | < 0.01 | N.D. | Isoproturon | < 0.01 | N.D. |
| Bentazone | < 0.01 | N.D. | Isoxaben | < 0.01 | N.D. |
| Biphenazate | < 0.01 | N.D. | Lambdacyhalothrin | < 0.01 | N.D. |
| Biterethanol | < 0.01 | N.D. | Malathion | < 0.01 | N.D. |
| Boscalid | < 0.01 | N.D. | Mecarbam | < 0.01 | N.D. |
| Bromacil | < 0.01 | N.D. | Mecoprop | < 0.01 | N.D. |
| Bromophosethyl | < 0.01 | N.D. | Mecoprop | < 0.01 | N.D. |
| Bromoxynil | < 0.01 | N.D. | Mepanipyrim | < 0.01 | N.D. |
| Bromoxynil | < 0.01 | N.D. | Mesosulfuronmethyl | < 0.01 | N.D. |
| Bromuconazole | < 0.01 | N.D. | Metalaxyl | < 0.01 | N.D. |
| Bupirimate | < 0.01 | N.D. | Metazachlor | < 0.01 | N.D. |
| Buprofezine | < 0.01 | N.D. | Methacriphos | < 0.01 | N.D. |
| Butacarboximsulfoxide | < 0.01 | N.D. | Methamidophos | < 0.01 | N.D. |
| Cadusafos | < 0.01 | N.D. | Method | < 0.01 | N.D. |
| Carbaryl | < 0.01 | N.D. | Metribuzin | < 0.01 | N.D. |
| Carbendazim | < 0.01 | N.D. | Mevinphos | < 0.01 | N.D. |
| Carbofuran | < 0.01 | N.D. | Molinate | < 0.01 | N.D. |
| Carbosulfan | < 0.01 | N.D. | Monocroptos | < 0.01 | N.D. |
| Carboxin | < 0.01 | N.D. | Monocroptos | < 0.01 | N.D. |
| Chlorfenvinphos | < 0.01 | N.D. | Monolinuron | < 0.01 | N.D. |
| Chlorfluazuron | < 0.01 | N.D. | Monuron | < 0.01 | N.D. |
| Chloridazone | < 0.01 | N.D. | Myclobutanil | < 0.01 | N.D. |
| Chloroxuron | < 0.01 | N.D. | Nuarimol | < 0.01 | N.D. |
| Chlorpropham | < 0.01 | N.D. | Omethoate | < 0.01 | N.D. |
| Chlorpyrifos | < 0.01 | N.D. | Oxadiargyl | < 0.01 | N.D. |
| Chlorpyrifos-methyl | < 0.01 | N.D. | Oxadiazone | < 0.01 | N.D. |
| Chlorsulfuron | < 0.01 | N.D. | Oxadixyl | < 0.01 | N.D. |
| Chlorthamide | < 0.01 | N.D. | Oxamyl | < 0.01 | N.D. |
| Cinidon-ethyl | < 0.01 | N.D. | Oxasulfuron | < 0.01 | N.D. |
| Clofentezine | < 0.01 | N.D. | Oxycarboxin | < 0.01 | N.D. |
| Cyazofamide | < 0.01 | N.D. | Oxyfluorfen | < 0.01 | N.D. |
| Cyclanilide | < 0.01 | N.D. | Penconazole | < 0.01 | N.D. |
| Cyloate | < 0.01 | N.D. | Pendimethalin | < 0.01 | N.D. |
| Cymoxanil | < 0.01 | N.D. | Permethrin | < 0.01 | N.D. |
| Cyproconazole | < 0.01 | N.D. | Pethoxamide | < 0.01 | N.D. |
| Cyprodinil | < 0.01 | N.D. | Phenarimole | < 0.01 | N.D. |
| Deltamethrin | < 0.01 | N.D. | Phenmedipham | < 0.01 | N.D. |
| Demetonsmethyl | < 0.01 | N.D. | Phenoxycarb | < 0.01 | N.D. |
| Demetonsmethylsulfoxide | < 0.01 | N.D. | Phenthoate | < 0.01 | N.D. |
| Desmedipham | < 0.01 | N.D. | Phorate | < 0.01 | N.D. |
| Diallate | < 0.01 | N.D. | Phosalon | < 0.01 | N.D. |
| Diazinon | < 0.01 | N.D. | Phosmet | < 0.01 | N.D. |
| Dichlofluanide | < 0.01 | N.D. | Phosphamidone | < 0.01 | N.D. |
| Dichlorvos | < 0.01 | N.D. | Phosthiasate | < 0.01 | N.D. |
| Difenoconazole | < 0.01 | N.D. | Picolinafen | < 0.01 | N.D. |
| Dimethenamide | < 0.01 | N.D. | Pirimiphos-methyl | < 0.01 | N.D. |
| Dimethoate | < 0.01 | N.D. | Primicarb | < 0.01 | N.D. |
| Dimethomorph | < 0.01 | N.D. | Prochloraz | < 0.01 | N.D. |
| Dimethomorph | < 0.01 | N.D. | Profenofos | < 0.01 | N.D. |
| Dimoxytrobin | < 0.01 | N.D. | Prometyrn | < 0.01 | N.D. |
| Diniconazole | < 0.01 | N.D. | Propamocarb | < 0.01 | N.D. |
| Dinocap | < 0.01 | N.D. | Propanyl | < 0.01 | N.D. |
| Dinoterb | < 0.01 | N.D. | Propargite | < 0.01 | N.D. |
| Diphenylamine | < 0.01 | N.D. | Propham | < 0.01 | N.D. |
| Disulfotonsulfone | < 0.01 | N.D. | Propiconazole | < 0.01 | N.D. |
| Disulfotonsulfoxide | < 0.01 | N.D. | Propyzamide | < 0.01 | N.D. |
| Disulphoton | < 0.01 | N.D. | Prothiophos | < 0.01 | N.D. |
| Dithalymphos | < 0.01 | N.D. | Pyraclostrobin | < 0.01 | N.D. |
| Dithianon | < 0.01 | N.D. | Pyrazophos | < 0.01 | N.D. |
| Diuron | < 0.01 | N.D. | Pyridaben | < 0.01 | N.D. |
| Dichlorprop | < 0.01 | N.D. | Pyridaphention | < 0.01 | N.D. |
| Epoxiconazole | < 0.01 | N.D. | Pyridate | < 0.01 | N.D. |
| Ethyl dipropylthiocarbamate | < 0.01 | N.D. | Pyrimethanil | < 0.01 | N.D. |
| Esfenvalerate | < 0.01 | N.D. | Pyriproxyfen | < 0.01 | N.D. |
| Ethiofencarb | < 0.01 | N.D. | Quinalphos | < 0.01 | N.D. |
| Ethion | < 0.01 | N.D. | Quinoxyfen | < 0.01 | N.D. |
| Ethofumesate | < 0.01 | N.D. | Quizalofopethyl | < 0.01 | N.D. |
| Ethoprophos | < 0.01 | N.D. | Resmethrin | < 0.01 | N.D. |
| Ethoxyquin | < 0.01 | N.D. | Rimsulfuron | < 0.01 | N.D. |
| Ethoxyquin | < 0.01 | N.D. | Simazine | < 0.01 | N.D. |
| Ethrimol | < 0.01 | N.D. | Spiroxamine | < 0.01 | N.D. |
| Etoxole | < 0.01 | N.D. | Taufluvalinate | < 0.01 | N.D. |
| Etrimphos | < 0.01 | N.D. | Tebuconazole | < 0.01 | N.D. |
| Fenamidone | < 0.01 | N.D. | Terbufos | < 0.01 | N.D. |
| Fenamiphosmt | < 0.01 | N.D. | Terbumetone | < 0.01 | N.D. |
| Fenbuconazole | < 0.01 | N.D. | Terbuthylazine | < 0.01 | N.D. |
| Fenhexamid | < 0.01 | N.D. | Terbutryn | < 0.01 | N.D. |
| Fenoxaprop-ethyl | < 0.01 | N.D. | Tetrachlorvinphos | < 0.01 | N.D. |
| Fenpropatrine | < 0.01 | N.D. | Thiacloprid | < 0.01 | N.D. |
| Fenpropimorph | < 0.01 | N.D. | Thifensulfuronmethyl | < 0.01 | N.D. |
| Fenthion | < 0.01 | N.D. | Thiodicarb | < 0.01 | N.D. |
| Fenvalerate | < 0.01 | N.D. | Thiophanatemethyl | < 0.01 | N.D. |
| Flazasulfuron | < 0.01 | N.D. | Tolclophos-methyl | < 0.01 | N.D. |
| Fluazifop-p-butyl | < 0.01 | N.D. | Tolylfluanide | < 0.01 | N.D. |
| Fludioxinil | < 0.01 | N.D. | Triadimenol | < 0.01 | N.D. |
| Fludioxonil | < 0.01 | N.D. | Triadimephone | < 0.01 | N.D. |
| Flufenacet | < 0.01 | N.D. | Triallate | < 0.01 | N.D. |
| Flufenoxuron | < 0.01 | N.D. | Triasulfuron | < 0.01 | N.D. |
| Fluopicolide | < 0.01 | N.D. | Trichlorphone | < 0.01 | N.D. |
| Flurochloridone | < 0.01 | N.D. | Tridemorph | < 0.01 | N.D. |
| Flusilazole | < 0.01 | N.D. | Trifloxystrobin | < 0.01 | N.D. |
| Flutolanil | < 0.01 | N.D. | Triflumizole | < 0.01 | N.D. |
| Furathiocarb | < 0.01 | N.D. | Triticonazole | < 0.01 | N.D. |
| Heptenophos | < 0.01 | N.D. |  |  |  |

*MRL—maximum residue level; N.D. Not detected.
